# Daylily intercropping: Effects on soil nutrients, enzyme activities, and microbial community structure

**DOI:** 10.3389/fpls.2023.1107690

**Published:** 2023-02-20

**Authors:** Jingxia Gao, Hua Xie

**Affiliations:** Ningxia Academy of Agriculture and Forestry Sciences, Yinchuan, Ningxia, China

**Keywords:** daylily, soil nutrients, enzymatic activity, microbial diversity, intercropping, yield

## Abstract

The daylily (*Hemerocallis citrina Baroni*)/other crop intercropping system can be a specific and efficient cropping pattern in a horticultural field. Intercropping systems contribute to the optimization of land use, fostering sustainable and efficient agriculture. In the present study, high-throughput sequencing was employed to explore the diversity in the root-soil microbial community in the intercropping of four daylily intercropping systems [watermelon (*Citrullus lanatus*)/daylily (WD), cabbage (*Brassica pekinensis*)/daylily (CD), kale (*Brassica oleracea*)/daylily (KD), watermelon/cabbage/kale/daylily (MI)], and determine the physicochemical traits and enzymatic activities of the soil. The results revealed that the contents of available potassium (2.03%-35.71%), available phosphorus (3.85%-62.56%), available nitrogen (12.90%-39.52%), and organic matter (19.08%-34.53%), and the urease (9.89%-31.02%) and sucrase (23.63%-50.60%) activities, and daylily yield (7.43%- 30.46%) in different intercropping soil systems were significantly higher compared to those in the daylily monocropping systems (CK). The bacterial Shannon index increased significantly in the CD and KD compared to the CK. In addition, the fungi Shannon index was also increased significantly in the MI, while the Shannon indices of the other intercropping modes were not significantly altered. Different intercropping systems also caused dramatic architectural and compositional alterations in the soil microbial community. A prominently higher relative richness of *Bacteroidetes* was noted in MI compared to that in CK, while *Acidobacteria* in WD and CD and *Chloroflexi* in WD were pronouncedly less abundant compared to those in CK. Furthermore, the association between soil bacteria taxa and soil characteristic parameters was stronger than that between fungi and soil. In conclusion, the present study demonstrated that the intercropping of daylily with other crops could significantly improve the nutrient levels of the soil and optimize the soil bacterial microflora composition and diversity.

## 1 Introduction

Daylily (*Hemerocallis citrina* Baroni.), common names Citron daylily and long yellow daylily, is a species of herbaceous perennial plant in the family Asphodelaceae, which is native to central and northern China, the Korea Peninsula, and Japan (Hou et al., 2017). Citron dayliy is now cultivated widely in Asia as ornamental plant and vegetable plant because of its beautiful flower, pleasant flavor, and beneficial secondary metabolites (Lin et al., 2013). In addition, daylily flowers has been used to treat various diseases including inflammation, insomnia, and depression (Yang et al., 2017). Due to the low cost of field management of day lily, one-time planting can benefit many years, and it has great economic benefits, so the cultivation area and output are constantly expanding. Growing demand for yields and the limited arable land have resulted in daylily production with high cropping intensity and monocultures over long periods (Zhu et al., 2018). Previous researches reported that continuous cropping obstacles often appear after a few years of continuous cropping (Xiong et al., 2015). Even with a good field management regime, crops under a continuous cropping system may still be affected by slower growth and development, lower yield and quality, and increased disease incidence (Li et al., 2016; Gao et al., 2019; Zeng et al., 2020).

Soil microorganisms are crucial for agricultural production as these contribute to maintaining soil quality and sustaining nutrient cycling (Sun et al., 2015; Beckers et al., 2017). The interaction between soil properties and the soil bacterial microflora affects the plant pathogens in certain cases, thereby influencing the health of plants (Wei et al., 2015). Therefore, management strategies related to the soil bacterial microflora have become an imperative research direction in the field of sustainable agriculture (Zhang et al., 2019). Intercropping refers to the concurrent cultivation of 2 crops in proximity. Intercropping, in addition to affecting crop yield, may also cause functional and architectural alterations in the soil microbiota (Duchene et al., 2017; Yu et al., 2018). In the intercropping systems, the soil microorganisms, enzymes, and nutrients may interact in various ways, which could either enhance or compromise the bacterial quantity and the enzymatic action, thereby facilitating or challenging the soil micro-ecotope improvement (Zhou et al., 2019). Soil microorganisms are crucial for facilitating various chemical, biological, and physical events in the soil, including the formation of the soil architecture, nutrient cycling, toxin aggregation/removal, organic matter turnover, and soil-borne pathogen inhibition (Bever et al., 2012; Blagodatskaya and Kuzyakov, 2013). Therefore, to elucidate the effects of intercropping on the soil microbiota architecture and diversity, enzyme activities, and nutrient levels, further research is warranted. Such research would be crucial for determining the energy output and nutrient status of the soil microorganisms in agroecosystems.

While the intercropping-induced alterations in soil microbial traits have been explored in a few studies (Jin et al., 2020), the alterations in the soil microbiota due to intercropping with other crops have not been studied in detail in the context of daylily. The diverse intercropping patterns exert different kinds of effects on the microbial characteristics and physicochemical traits of the soil. Therefore, in the present study, the bacterial and fungal microflora alterations occurring in the intercropping systems comprising daylily with various other crops were assessed. It was hypothesized that intercropping would greatly impact the soil physicochemical traits, enhance the microbiota diversity and enzyme activities, and alter the architecture of the soil microbiota. The main objectives of the present study were as follows: (1) investigating the effects of daylily intercropping with various other crops on the soil physicochemical properties, soil enzyme activities, and daylily yield; (2) comparing the differences in the bacterial and fungal diversity and the soil microflora composition between monocropping and intercropping; and (3) determining the associations of soil bacterial microflora with the enzymatic activities and physicochemical traits of the soil.

## 2 Materials and methods

### 2.1 Experimental design and sampling

The experiment was conducted at the Modern Agricultural Plantation of the Ningxia Academy of Agriculture and Forestry, Yinchuan, Ningxia, China (38°28’48” N, 106°13’31” E; altitude 1100 m). The field soil was sand-loam, and a 3-year continuous daylily cropping was implemented for the plantation. The climate of the temperate continental was semi-arid kind with the average yearly temperature (8.5°C), precipitation (200 mm), potential precipitation (2250 mm), and sunshine (2850 h), respectively. The frost-free season lasted for 185 days.

Watermelon (*Citrullus lanatus*), cabbage (*Brassica pekinensis*), and kale (*Brassica oleracea*) were intercropped with daylily. The experimental design included the following 5 treatments: (1) watermelon–daylily intercropping (WD); (2) cabbage–daylily intercropping (CD); (3) kale–daylily intercropping (KD); (4) row mixed intercropping in which watermelon, cabbage, and kale were alternately intercropped with daylily (MI); (5) monoculture of daylily (CK). A completely randomized design was adopted for field experimentation, with 3 plots (each with a surface area of 120 m^2^) per treatment. Daylily was planted on April 16, 2020, and later, on May 20, 2020, watermelon, cabbage, and kale were intercropped with daylily. The planting densities of different treatments are presented in **Figure S1**. Drip irrigation was used for all crops, and the entire cultivation was managed conventionally.

The soil near the roots of daylily were collected from 15 test plots using an auger on August 30, 2020. After the random collection of 5 soil sub-samples (10–20 cm depth) from each experimental plot, all samples were integrated into a whole soil sample. Prior to laboratory transfer, this whole soil sample was sieved through a 2-mm mesh for complete homogenization and elimination of stones, roots, and plant residues. Each sample was divided into 2 aliquots, one of which was stored at an ambient temperature for use in the chemical assay while the other was cryopreserved at –80°C for later use in the biological analysis. The soil samples for chemical analysis were cryopreserved at –20°C after a week of air-drying treatment.

### 2.2 Soil properties and enzyme activities

Soil pH was determined using a pH meter (PHS-25, Shanghai, China) at a constant soil/water ratio of 1:2.5. The available nitrogen (AN) concentration in the soil samples was determined using the DigiPREP TKN System (KJELTEC 8400, Foss, Denmark). A UV–Vis spectrophotometer (UH5300, North Points Ruili) was utilized for assessing the available phosphorus (AP) concentration in the soil. The available potassium (AK) in the soil was quantified using an inductively coupled plasma (ICP) spectrometer (Spectro Analytical Instruments, Kleve, Germany). The K_2_Cr_2_O_7_-H_2_SO_4_ oxidation approach was adopted to assess the organic matter (OM) content in the soil.

Soil enzymes associated with nitrogen, phosphorus, and carbon degradation, including peroxidase (POD), sucrase (SC), and urease (UE), were evaluated for their activity. The soil UE activity was determined as described by Bao (2000) (Bao, 2000) using urea as the substrate. The spectrophotometric approach was adopted for the soil POD quantification in a 96-well microplate, using L-3,4-dihydroxyphenylalanine (L-DOPA) as the substrate (Bach et al., 2013). The soil SC activity was evaluated by determining the glucose discharge from an 8% sucrose solution following 24 h of incubation at 37°C (Chen et al., 2010).

### 2.3 Soil DNA extraction, PCR amplification, and sequencing

The total genomic DNA was extracted from the soil samples using sterile cotton swabs and the FastDNA spin kit for soil (MP, USA) according to the provided protocol. The extracted genomic DNA was amplified and sequenced by the Gene Denovo company. Li and Wu’s report (2018) was referred to for designing the specific primers ITS4–2409R and ITS3_KYO2F for the ITS2 zone amplification of the ITS rRNA gene, and the specific primers 341F and 806R for the V3-V4 zone amplification of the 16S rRNA gene (Li and Wu, 2018). In order to sequence the purified amplicons, paired-end sequencing (HiSeq 2500, PE250) was performed on the Illumina platform following standard operations.

Quality filtration and sequence fusion were conducted on the raw fastqfiles of 50 microbial samples using FASTP version 0.18.0 and FLSAH version 1.2.11, thereby deriving Tags. The next step was to filter out the low-quality tags following the Tags Quality Control process of QIIME Ver. 1.9.1, which generated high-quality Clean Tags (Edgar, 2013). The chimeras of the tags were recognized and deleted *via* the UCHIME algorithm by exploiting the reference database, thereby generating valid Clean Tags (Edgar et al., 2011). Finally, using UPARSE version 9.2.64, the Clean Tags were assigned to the identical Operational Taxonomic Units (OTUs) in the similarity setting of tingil (Edgar, 2013). In this process, for every OUT, the tag sequence with the maximum richness was selected as the typical sequence, and for every typical sequence, the classification information was annotated *via* the Mothur algorithm in the Silva database (Quast et al., 2013).

### 2.4 Statistical analyses

The “Vegan” R software version 2.5–6 was employed for estimating the alpha- and beta-diversities (Bray–Curtis dissimilarity). SPSS 25.0 (IBM, USA) was used for conducting the one-way analysis of variance (ANOVA) and the least significant difference (LSD) approach (*p* < 0.05) to determine whether the differences in the inter-sample diversities based on the alpha index were statistically significant. The distribution trends of the soil microorganism communities in different treatments were assessed using the ‘labdsv’ package for the principal coordinates analysis (PCoA). Analysis of similarities (ANOSIM) and permutational multivariate analysis of variance (ADONIS) with 999 permutations were used for determining the significant inter-sample group differences in the beta diversity using Bray–Curtis distance matrices. The taxonomic traits of the microbial species characterizing the inter-treatment differences (https://www.cloudtutu.com) were elucidated through the linear discriminant analysis effect size (LEfSe) assay. In order to simplify the LEfSe process, only the OTUs with a relative richness of >0.01% were selected through OTU table filtration screened. The taxa with significant differential inter-group richness levels were recognized using the factorial Kruskal–Wallis sum-rank test (α = 0.05). Thereafter, the effect size was computed for every discriminative trait based on the logarithmic LDA score (threshold = 2.0). The associations of the environmental parameters with the microbial communities were explored using the “vegan” R software for redundancy analysis (RDA) and the Mantel test. Using the same”vegan” package, the associations of the OTUs with the physicochemical properties and enzymatic activities were visualized through RDA.

## 3 Results

### 3.1 Effects of intercropping on the soil physicochemical properties and enzyme activities

Among all treatments, prominently higher levels of AN, AK, AP, and OM (LSD, *p* < 0.05, **Table 1**) were noted in the intercropping soil systems (WD, CD, KD, and MI) compared to that in the monocropping system (CK). The nutrient levels were higher in WD compared to CK by 12.90% for AN, 15.8% for AP, 35.71% for AK, and 30.95% for OM. The nutrient levels were higher in CD compared to CK by 39.52% for AN, 62.56% for AP, 26.47% for AK, and 34.53% for OM. The nutrient levels were higher in KD compared to CK by 17.21% for AN, 35.90% for AP, 14.90% for AK, and 20.46% for OM. The nutrient levels were higher in MI compared to CK by 31.73% for AN, 3.85% for AP, 2.03% for AK, and 19.08% for OM (**Table 1**). Among the different intercropping soil systems, CD presented the highest concentrations of soil AN, AP, and OM, while WD presented the highest soil AK concentration (**Table 1**). The WD, CD, and KD systems were less different from CK in terms of soil pH while presenting significantly increased MI (**Table 1**). Similarly, soil enzyme activities differed significantly. The UE and SC activities were significantly higher in the intercropping soil systems (WD, CD, KD, and MI) compared to the monocropping soil system (CK) (**Table 1**). The POD activity was significantly higher in WD and MI compared to CK, while it was significantly lower in CD and KD (**Table 1**). For yield, the yield of daylily in all intercropping soil systems was higher than that in monocropping systems, with WD having the highest yield (30.46%), followed by MI (18.35%), CD (12.81%) and KD (7.43%) (**Table 1**).

**Table 1:** Chemical properties and enzymatic activities of soil.

	WD	CD	KD	MI	CK	*P*-values	significant
pH	7.90 ± 0.01b	7.91 ± 0.01b	7.90 ± 0.02b	7.95 ± 0.02a	7.91 ± 0.01b	0.004	**
OM (g/kg)	20.86 ± 0.93ab	21.43 ± 1.79a	19.19 ± 0.77b	18.97 ± 1.11b	15.93 ± 0.45c	0.001	**
AN (mg/kg)	70.00 ± 1.01c	86.5 ± 1.49a	72.67 ± 1.53c	81.67 ± 1.52b	62.00 ± 1.05d	0	**
AP (mg/kg)	156.33 ± 0.77a	211.33 ± 0.42c	176.67 ± 0.63b	135.00 ± 0.43d	130.00 ± 0.35e	0	**
AK (mg/kg)	79.70 ± 0.58c	75.78 ± 2.89a	68.85 ± 3.06b	59.92 ± 1.01d	58.73 ± 1.02e	0	**
UE (U/g)	73.80 ± 0.25c	70.14 ± 0.59d	83.63 ± 0.53a	80.08 ± 0.58b	63.83 ± 2.08e	0	**
POD (U/g)	42.24 ± 0.47a	37.97 ± 0.59d	39.45 ± 0.55c	41.81 ± 0.19a	40.71 ± 0.16b	0	**
SC (U/g)	241.45 ± 1.55c	293.29 ± 1.38a	257.91 ± 1.91b	294.12 ± 1.29a	195.3 ± 1.41d	0	**
Yield (kg/ha)	21,553 ± 113a	18,637 ± 125c	17,749 ± 131d	19,553 ± 113b	16,521 ± 182e	0	**

Values are presented as mean ± standard error (n = 3). Different lowercase letters indicate statistically significant differences (P < 0.05) by Tukey’s test between different treatments. **, indicates a significant difference at the P < 0.01 level. AN, available nitrogen; AP, available phosphorus; AK, available potassium; OM, organic matter. UE, urease; POD, peroxidase; SC, sucrase. WD, watermelon-daylily intercropping; CD, cabbage-daylily intercropping; KD, kale-daylily intercropping; MI, row mixed intercropping, watermelon, cabbage, and kale were alternately intercropped with daylily; CK, monoculture daylily.

### 3.2 Effects of intercropping on microbial community diversity and community structure

**Figure 1:**
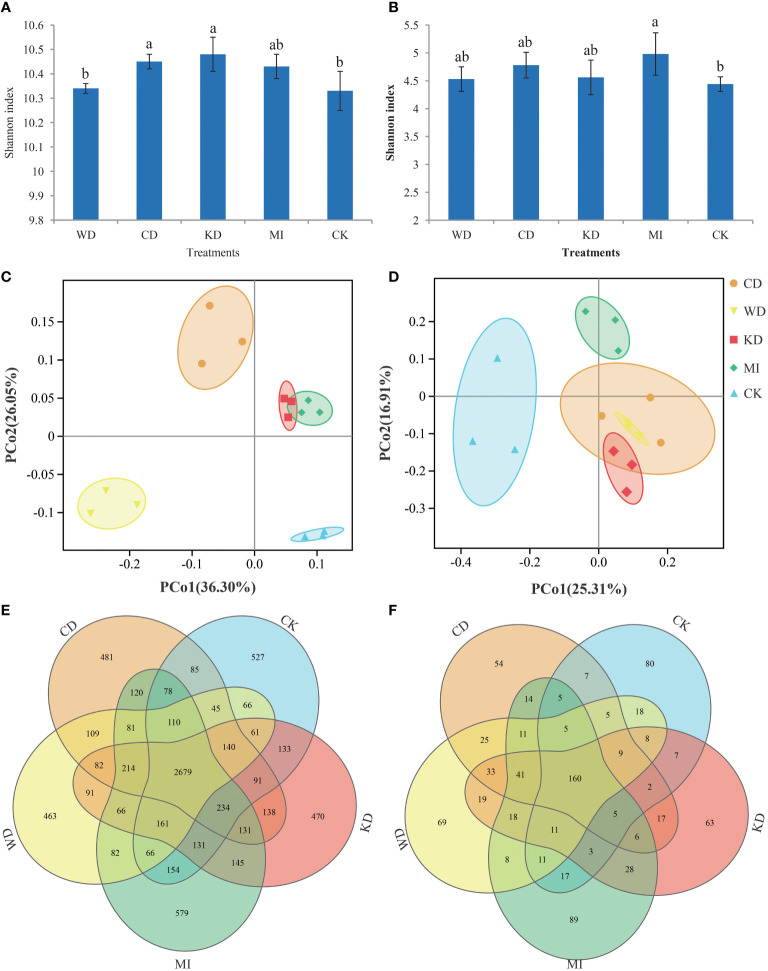
Microbial community diversity in different intercropping modes. Shannon index diversity of bacteria **(A)** and fungi **(B)** communities under different treatments. A lowercase letter on each box represents a least significant difference (LSD; *p* < 0.05) between treatments. Principal coordinate analysis (PCoA) plots based on the Bray-Curtis distance demonstrating the separation between soil bacteria **(C)** and fungi **(D)** communities of different treatments. The Venn diagram shows the numbers of bacteria **(E)** and fungi **(F)** operational taxonomic units (OTUs) that are shared or unshared by different treatments. WD, watermelon-daylily intercropping; CD, cabbage-daylily intercropping; KD, kale-daylily intercropping; MI, row mixed intercropping, watermelon, cabbage, and kale were alternately intercropped with daylily; CK, monoculture daylily.

Approximately 1,665,552 superior-quality V3-V4 sequences and 1,501,148 superior-quality ITS2 sequences were yielded in total in the raw-read sequencing process. The mean read lengths for bacteria and fungi were 456 bp and 357 bp, respectively. The sequences of all samples were clustered into 93,898 bacterial OTUs and 8,372 fungal OTUs, using an identity threshold of 97%. The flat trend of the rarefaction curve (**Figure S2**) suggested that the desired overall coverage of OTUs was provided by deep sequencing. **Figure 1** depicts the calculated alpha diversity (α-diversity) indices of the bacterial and fungal communities for the different intercropping modes based on the Shannon index metric, which incorporates both the abundance and evenness of the microbiota. In the case of the bacterial microflora, the Shannon diversity values were in the following order: KD > CD > MI >WD > CK; in comparison to CK, the Shannon diversity values of CD and KD were pronouncedly higher (LSD, *p* < 0.05, **Figure 1A**), while no significant differences (*p* > 0.05, **Figure 1A**) were observed among WD, MI, and CK. In the case of the fungal microflora, the Shannon diversity values were in the following order: MI > CD > KD > WD > CK; in comparison to CK, the Shannon index of MI was pronouncedly higher (*p* < 0.05, **Figure 1B**), while no significant differences were observed among CD, KD, WD, and CK (*p* > 0.05, **Figure 1B**). The microbiota differences among the different treatments were assessed using the Bray-Curtis distance-based principal coordinate analysis (PCoA). In the case of bacterial microbiota, a sharp separation was observable among CD, WD, KD, MI and CK, while KD and MI were partially overlapped (**Figure 1C**); as demonstrated by the PCoA outcomes, the first 2 axes explained 36.3% and 26.05%, respectively, of the overall variation in the bacterial microflora. In the case of fungal microbiota, a distinct separation was observed among KD, WD, MI, and CK, while CD was overlapped with both KD and WD (**Figure 1D**); the PCoA outcomes revealed that the first 2 axes explained 25.31% and 16.91%, respectively, of the overall variation in the fungal microflora. The results of ANOSIM and ADONIS also indicated significant differences in the bacterial and fungal microbiota in the soil between a minimum of two treatments (**Table S1**). The Venn diagrams verified that the variation in the microflora among different soil intercropping systems was due to the alterations in the composition of several unique and shared OTUs. Among all the OTUs identified in this study, the bacteria shared across all intercropping soil systems had 2679 OTUs, the WD had 463 unique OTUs, the CD had 481 unique OTUs, the KD had 470 unique OTUs, the MI had 579 unique OTUs, the CK had 527 unique OTUs (**Figure 1E**). Among all the OTUs identified in this study, the fungi shared across all intercropping soil systems had 160 OTUs, the WD had 69 unique OTUs, the CD had 54 unique OTUs, the KD had 63 unique OTUs, the MI had 89 unique OTUs, the CK had 80 unique OTUs (**Figure 1F**).

### 3.3 Effects of intercropping on the composition of soil microbiota

**Figure 2:**
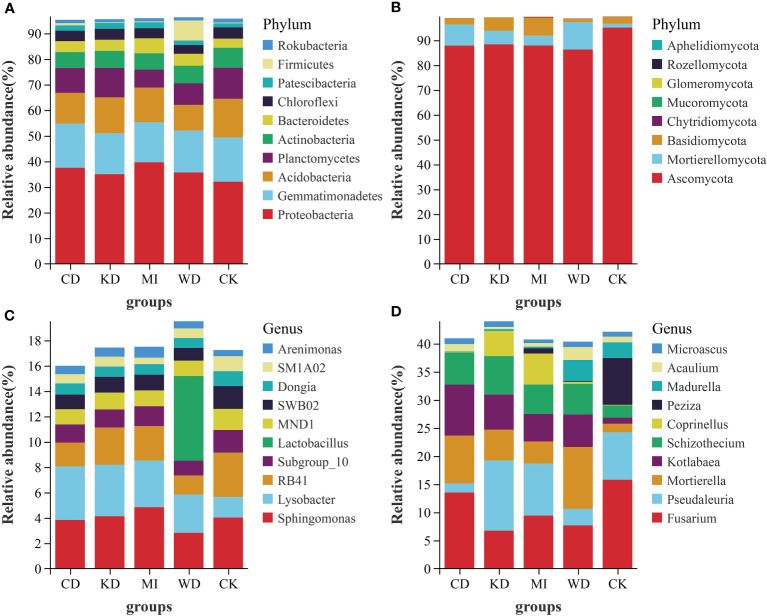
Relative abundance at phylum and genus level of soil microbial community of intercropping systems (top 10 shown). **(A)** bacteria phylum level; **(B)** fungi phylum level; **(C)** bacteria genus level; **(D)** fungi genus level. WD, watermelon-daylily intercropping; CD, cabbage-daylily intercropping; KD, kale-daylily intercropping; MI, row mixed intercropping, watermelon, cabbage, and kale were alternately intercropped with daylily; CK, monoculture daylily.

**Figure 2** depicts the outcomes of the sequence analyses conducted at the genus and phylum levels. In the case of bacteria, the richest phylum was *Proteobacteria* (35.93%), followed by *Gemmatimonadetes* (16.51%) and *Acidobacteria* (12.99%), respectively. Among the dominant taxa, the abundances of *Proteobacteria*, *Gemmatimonadetes*, *Planctomycetes*, *Actinobacteria*, *Patescibacteria*, and *Firmicutes* did not change significantly in the intercropping soil systems (CD, WD, KD, and MI) compared to their abundances in the monocropping soil system (CK) (**Table S2**, **Figure 2A**). *Rokubacteria* in KD and *Acidobacteria* in WD and CD were significantly less abundant compared to CK. A significantly greater abundance of *Chloroflexi* in WD and *Bacteroidetes* in MI was observed compared to CK (**Table S2**, **Figure 2A**). The richest genera in the different intercropped soils were *Sphingomonas* (3.94%), *Lysobacter* (3.33%), RB41 (2.51%), Subgroup*_*10 (1.49%), *Lactobacillus* (1.34%), MND1 (1.33%), SWB02 (1.29%), *Dongia* (0.90%), SM1A02 (0.80%), and *Arenimonas* (0.67%) (**Figure 2C**, **Table S2**). *Sphingomonas*, MND1, SWB02, *Dongia*, and *Arenimonas* were not significantly altered in the intercropping systems (WD, CD, KD, and MI) compared to the monocropping system (CK) (**Table S2**). A significantly greater abundance of Lysobacter in CD, KD, and MI and that of Lactobacillus in WD was observed compared to CK. RB41 in WD and CD, Subgroup*_*10 in WD, and SM1A02 in MI were significantly less abundant compared to CK (**Table S2**).

In the case of fungi, the richest phylum was *Ascomycota* (89.05%), followed by *Mortierellomycota* (6.11%) and *Basidiomycota* (3.88%). Among the dominant taxa, the abundances of *Ascomycota*, *Chytridiomycota*, *Glomeromycota*, *Rozellomycota*, and *Aphelidiomycota* did not change significantly in the 4 investigated intercropping systems compared to those in the monocropping soil system (CK) (**Table S3**, **Figure 2B**). A significantly greater abundance of *Mortierellomycota* in WD and CD, *Basidiomycota* in KD and MI, and *Mucoromycota* in CD was observed compared to CK (**Table S3**, **Figure 2B**). The richest genera in the different intercropping soil systems were *Fusarium* (10.65%), *Pseudaleuria* (6.92%), *Mortierella* (6.11%), *Kotlabaea* (5.42%), *Schizothecium* (5.06%), *Coprinellus* (2.11%), *Peziza* (1.88%), *Madurella* (1.46%), *Acaulium* (1.13%), and *Microascus* (0.92%) (**Figure 2D**, **Table S3**). *Kotlabaea* and *Schizothecium* were prominently richer, while *Fusarium* and *Peziza* were pronouncedly less abundant in the 4 investigated intercropping systems compared to their abundances in the monocropping soil system (CK); the differences in the abundance of *Microascus* were not significant between the two kinds of systems (**Table S3**). Significantly greater abundances of *Mortierella* in WD, CD, and KD, *Pseudaleuria* in KD, *Coprinellus* in KD and MI, and *Madurella* and *Acaulium* in WD were observed compared to CK. Significantly lower abundances of *Pseudaleuria* in WD and CD and *Madurella* in CD, KD, and MI were observed compared to CK (**Table S3**).

**Figure 3:**
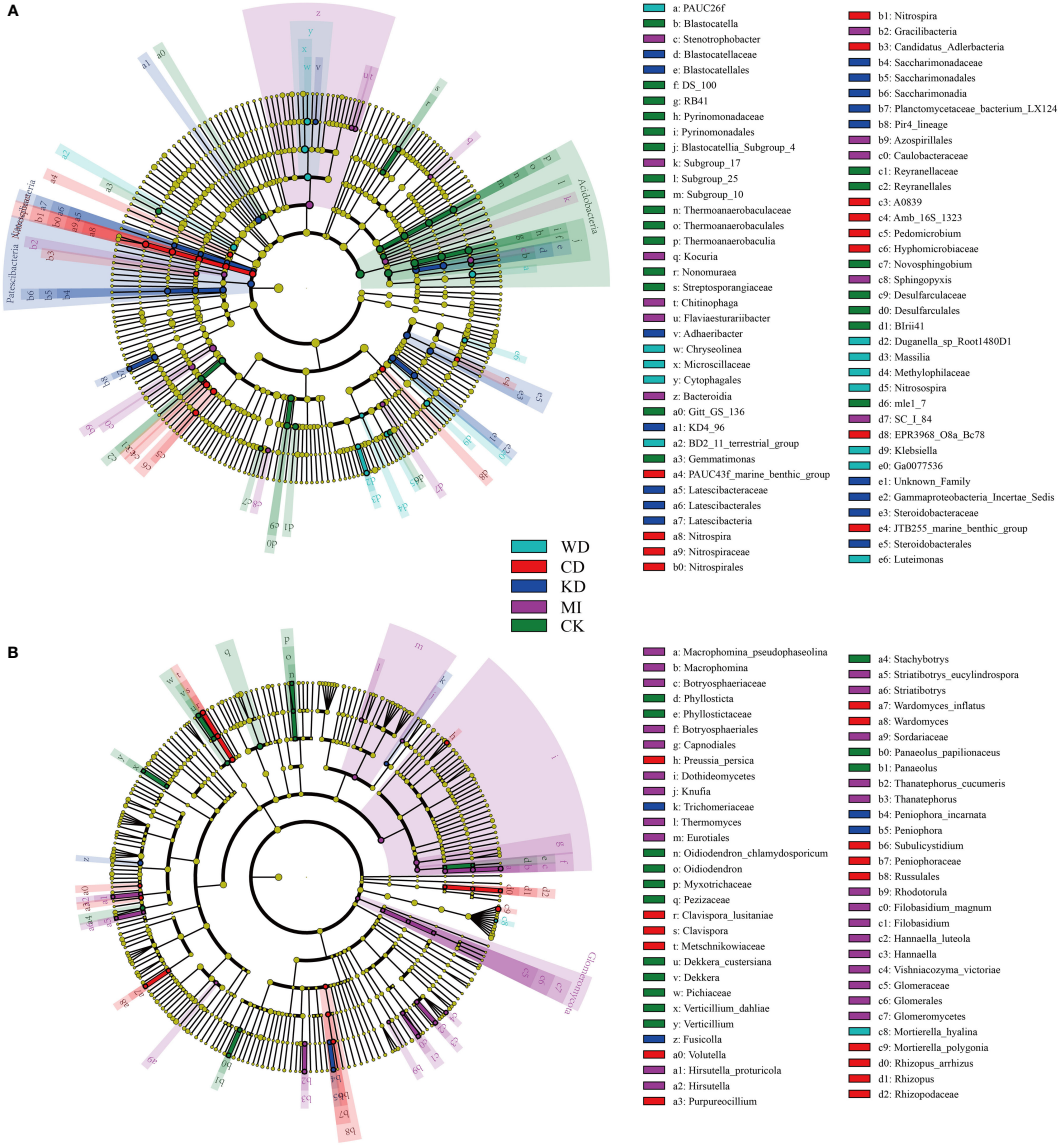
Linear discriminant analysis effect size (LEfSe) taxonomic cladogram identified significantly discriminant bacteria **(A)** and fungi **(B)** taxa (LDA > 2) associated with soils of different intercropping modes. WD, watermelon-daylily intercropping; CD, cabbage-daylily intercropping; KD, kale-daylily intercropping; MI, row mixed intercropping, watermelon, cabbage, and kale were alternately intercropped with daylily; CK, monoculture daylily.

Further, the effects of the different intercropping patterns on soil microbial community composition were determined using the linear discriminant analysis effect size analysis (LEfSe). LEfSe results revealed seventy-seven bacterial taxa that differed significantly in terms of relative richness among the different intercropped soil systems. As depicted in **Figure 3A**, 12 bacterial taxa in WD, 18 bacterial taxa in CD, 13 bacterial taxa in KD, 11 bacterial taxa in MI, and 23 bacterial taxa in CK were identified as biomarkers. As revealed by LEfSe, 60 fungal taxa differed significantly in terms of relative richness among the different intercropped soil systems. Among the identified biomarkers were one fungal taxon in WD, 4 fungal taxa in CD, 15 fungal taxa in KD, 26 fungal taxa in MI, and 14 fungal taxa in CK (**Figure 3B**).

### 3.4 Relationships between the soil properties and the soil microbial communities

**Figure 4:**
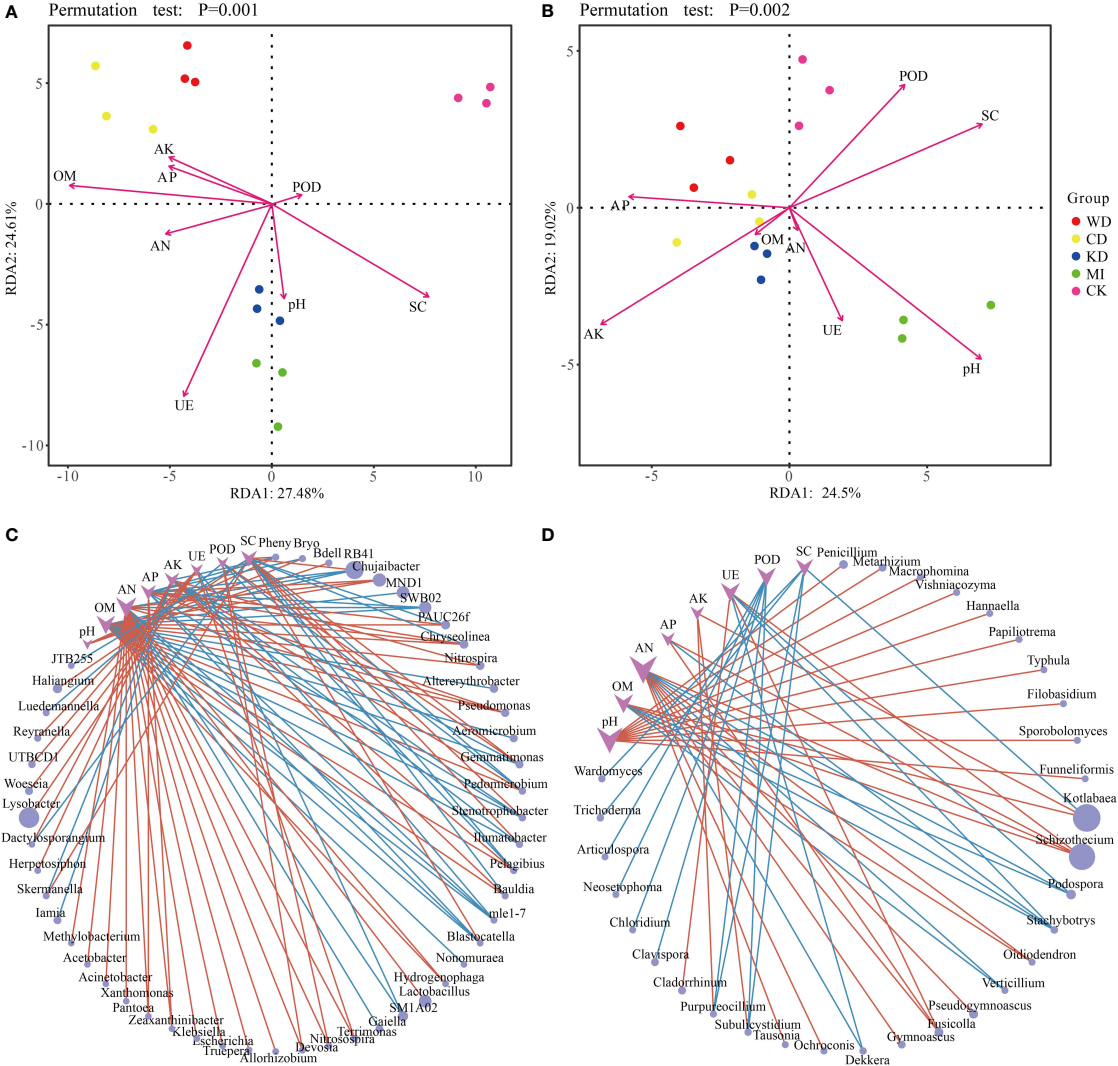
Statistical correlation of soil properties with the microbiome. Redundancy analysis (RDA) based on bacteria **(A)** and fungi **(B)** community composition of intercropping systems. Network analysis reflected the co-occurrence relationship between bacteria **(C)** and fungi **(D)** taxa and soil parameters. Purple vee nodes represent soil parameters. Grey ellipse nodes represent microbial members. Direct connections between nodes indicate strong correlations (Pearson correlation coefficient, ∣r∣ ≥ 0.5; P < 0.05). The color of the edges represents positive correlation (pink) or negative correlation (blue). The sizes of vee nodes are proportional to the interconnected degree. The sizes of ellipse nodes are proportional to the relative abundance. AN, available nitrogen; AP, available phosphorus; AK, available potassium; OM, organic matter. UE, urease; POD, peroxidase; SC, sucrase. WD, watermelon-daylily intercropping; CD, cabbage-daylily intercropping; KD, kale-daylily intercropping; MI, row mixed intercropping, watermelon, cabbage, and kale were alternately intercropped with daylily; CK, monoculture daylily.

Redundancy analysis (RDA) revealed a relationship between the microbial community structure, soil properties (soil chemistry and enzymatic activity) and daylily yield. In the case of bacteria, AK, AP, AN, and OM were closely associated with WD and CD; UE, pH, and SC were closely related to KD and MI; POD was closely related to CK; Yield was closely related to WD and CD, and was also affected by AK, AP, AN and OM (**Figure 4A**). The ANOVA-like permutation test revealed that OM, UE, SC, AN, AK, Yield, and AP were significantly associated with the bacterial communities (**Table S4**, p-value < 0.01), while pH and POD did not exhibit a significant association (**Table S4**, p-value > 0.05). In the case of fungi, AP, AK, and OM were closely related to WD, CD, and KD; AN, UE, and pH were closely related to MI; POD and SC were closely related to CK; Yield was closely related to WD, CD and KD, and was also affected by AK, AP, AN and OM (**Figure 4B**). The ANOVA-like permutation test revealed that AP, AK, and SC were significantly associated with the fungal communities (**Table S4**, p-value < 0.05), while pH, OM, AN, UE, POD, and Yield did not exhibit a significant association (**Table S4**, p-value > 0.05).

The co-occurrence patterns between the microbiome and soil properties were investigated using network analysis. As depicted in **Figures 4C, D**, overall, the module was more densely connected in the case of bacteria (average degree = 1.72) compared to fungi (average degree = 1.28). Several microbial taxa were associated significantly with various soil parameters, particularly AN, which exhibited high connectivity. For instance, Blastocatella was linked positively to SC and negatively to AN, AP, and OM, Gemmatimonas was associated positively with SC and POD and negatively with OM and AK (**Figure 4C**), Fusicolla was associated positively with AN, AP, and UE, and Stachybotrys was associated negatively with OM, AN, AP, and UE (**Figure 4D**).

## 4 Discussion

Intercropping is considered to be an environmentally friendly system that can improve crop yield as well as water and nutrient-use efficiency (Gaiser et al., 2004; Li et al., 2014; Cong et al., 2015; Dai et al., 2019). One of the greatest superiorities of intercropping is a further efficient utilization of lands and positive inter-crop interplays, which facilitates the survival, fitness, and growth of crops (Hauggaard-Nielsen and Jensen, 2005). Intercropping systems are reported to improve the carbon and nitrogen concentrations, physicochemical traits, bulk density, and pH of the soil (Bedoussac and Justes, 2009; Liu et al., 2014; Li and Wu, 2018; Cuartero et al., 2022). The present study also verified that the levels of nutrients (AP, AN, AK, and OM) in the soil and daylily yields were prominently higher in all intercropping systems compared to the monocropping system, suggesting that concurrent growth of 2 or more crops could contribute to elevating the levels of soil nutrients (**Table 1**). The soil nutrient contents and daylily yields of the different intercropping soil systems (WD, CD, KD, and MI) were significantly different, indicating the existence of crop competition for soil nutrients. For instance, a pronouncedly lower nitrogen concentration in WD was noted compared to that in CD, KD, and MI (**Table 1**). This could be related to the development of watermelon, as substantial nitrogen is necessary for promoting photosynthesis and, ultimately, the watermelon vine growth, thereby affecting the watermelon quality (Gulut, 2021). The contents of AK and AP were significantly increased in the intercropped soils, which may be attributed to the increased richness of the soil nutrient cycling-associated microorganic taxa, such as phosphate- and potassium-solubilizing bacteria and rhizobia (Palm, 1995). This increase in yield could be due to higher nitrogen disposal from the rhizosphere of intercropping systems, which should be higher in soils with low N fertilization addition (Yu et al., 2018). This fact has previously been observed in other intercrop relationships, such as cowpea-maize (Latati et al., 2014).

Soil enzymatic activity is a vital parameter indicating how organic matter is degraded, and the nutrients are cycled in the soil (Nannipieri et al., 2012; Hussain et al., 2021). Soil enzymatic activity is reportedly impacted by the physicochemical traits and microbial communities of the soil (Gu et al., 2009). In the present study, the intercropping soil systems (WD, CD, KD, and MI) presented the highest UE and SC activities compared to the CK. Bai et al. (2022) also reported that the sucrase activity of the soil in the intercropping forests was higher than that in the monocropping walnut and tea forests. Zhou et al. (2011) reported elevation in the soil urease activity in the cucumber–garlic/onion intercropping system, with this effect persisting a few growing seasons compared to the cucumber monocropping system. Sucrase hydrolyzes sucrose and, therefore, reflects the convertibility of the soil organic carbon, while urease impacts the metabolism of soil nitrogen through urea hydrolyzation (Cantarella et al., 2018). Peroxidase activity is considered a crucial predictor of the dynamics of soil organic matter (Tian and Shi, 2014). In the present study, the intercropping soil systems (WD, CD, KD, and MI) presented weakened POD activity as the availability of inorganic nitrogen increased (**Table 1**), which is consistent with the results reported by Bai et al. (2022). It appears that different intercropping patterns of different crops play a pivotal role in the aggregation, decomposition, and conversion of the soil organic carbon, and are, therefore, capable of offering sufficient energy to the soil microbes.

Soil microorganic diversity is strongly associated with the stability of the soil ecotope and soil nutrient conversion, and intercropping facilitates the management of diverse agroecosystem services through the upgradation of soil quality (Cong et al., 2015). It is noteworthy that the composition of soil microbiota is linked remarkably to the alterations in the soil chemical traits (Lauber et al., 2008; Campbell et al., 2010). In the present work, intercropping systems presented improved microbial diversity and nutrient content in the soil (**Figures 1A, B**; **Table 1**), suggesting that besides enhancing the quality of the continuous cropping soil, intercropping also facilitates enhancing the stability of the facility ecosystem. The potential factors that influence the soil microbiota include the soil types, plant varieties, and root exudates (Wieland et al., 2001; Broeckling et al., 2008; Lauber et al., 2008; Lauber et al., 2009). Significant alterations in the composition of the soil microorganic taxa were noted among the monocropping and intercropping systems in the present study (**Figures 2C, D**). For instance, both unique OTUs and marker taxa in the different intercropping soil systems (WD, CD, KD, and MI) were altered compared to CK (**Figure 1E, F**; **Figure 3**). The prevailing taxonomic groups identified in the investigated soils were *Proteobacteria*, *Actinobacteria*, *Acidobacteria*, *Firmicutes*, *Gemmatimonadetes*, *Planctomycetes*, *Chloroflexi*, and *Bacteroidetes*, all of which are ordinary soil inhabitants (Zhou et al., 2018), and the same result was also observed in this study. As implied by a higher relative richness of *Bacteroidetes* in MI and the lower richness of *Acidobacteria* in WD and CD and that of *Chloroflexi* in WD, compared to CK (**Table S2**), the richness of the prevailing taxa in the soil is alterable through changes in both planting patterns and crop species, as since these taxa are adaptable to a novel microenvironment (Zhang et al., 2018; Gong et al., 2019; Zhi-dan et al., 2019).

Further, the different intercropping modes led to alterations in the soil physicochemical traits and enzymatic activities, prompting the enrichment of a particular subset of functional bacteria and fungi in the soil, which manifested as elevated diversities of both microbial types in the intercropped soil compared to the tea monoculture (Bai et al., 2022). Previous reports have indicated that *Bacteroidetes* are linked to the cycling of N and P in soil (Lidbury et al., 2021). A few plant-beneficial microbes recognized in previous studies, such as *Bacillus*, *Pseudomonas*, *Sphingomonas*, and *Streptomyces* (Bhattacharyya and Jha, 2012; Asaf et al., 2020), were reported to be capable of lowering the proportion of harmful fungi in the soil (Negawo and Beyene, 2017) through the inhibition of fungal growth and facilitating plant growth (Tejera-Hernández et al., 2011; Sivasakthi et al., 2014). In the present study, the richness of *Pseudomonas* exhibited a significant positive association with AP, AN, and OM (**Figure 4C**). In addition, the richness of *Chujaibacter*, a bacteria implicated in nitrification (a process of N cycling) (Semenov et al., 2020), was linked positively to the levels of AP, AN, and OM in the soil (**Figure 4C**). Improvement in the uptake of nitrogen and phosphorus in the presence of *Oidiodendron* has been reported previously (Vohník et al., 2005). In the present study, the richness of *Oidiodendron* exhibited a significant positive association with OM and AN (**Figure 4D**). In addition, the different intercropping soil systems (WD, CD, KD, and MI) presented the enrichment of a few bacterial genera related to potassium and phosphorus solubilization. Examples of these bacterial genera include *Pseudomonas*, *Arthrobacter*, *Sphingomonas*, *Bacillus*, and *Burkholderia* (data not presented), which have been previously acknowledged to possess functions such as facilitation of plant growth, solubilization of phosphorus, fixation of nitrogen, organic compound degradation and bioconversion, and stimulation of growth (Rodríguez and Fraga, 1999; Sharma et al., 2013; Panhwar et al., 2014; Chen et al., 2016; Sadauskas et al., 2020; Tapia-García et al., 2020; Yang et al., 2020). Similarly, the potassium-solubilizing activities of *Bacillus* and *Burkholderia* have also been reported previously (Basak and Biswas, 2008; Zhang and Kong, 2014). These results indicated that intercropping impacts the soil nutrient levels, microbiota composition, and enzymatic activity favorably and that microorganisms have crucial roles to play in nutrient cycling.

## 5 Conclusion

Intercropping of daylily with other crops could increase the concentrations of organic matter, nitrogen, potassium, and phosphorus in the soil and daylily yield, thereby facilitating the improvement of soil nutrient status greatly. Meanwhile, the different intercropping modes also caused great alterations in the architecture and diversity of the microbial communities in the soil. In addition, the microbial taxa in the soil were closely related to soil characteristic parameters. In conclusion, besides significantly optimizing the microbiota architecture in the soil, the intercropping system also led to an enhanced relative abundance of the beneficial taxa of bacteria and fungi associated primarily with disease prevention and nutrient cycling. Further long-term analysis of these intercropping systems will be needed to reinforce findings on the positive interaction between daylily and other crops microbiota and their functions, and to study more in depth which intercropping pattern would be the most beneficial for the farmer and contribute to the development of sustainable agriculture.

## Data availability statement

The data presented in the study are deposited in the CNCB repository (https://ngdc.cncb.ac.cn/databases), accession number PRJCA010600.

## Author contributions

JG performed the statistical analysis and wrote the first draft of the manuscript. HX contributed to conception and design of the study. All authors contributed to manuscript revision, read, and approved the submitted version.
