# Supplementary material for: Daylily intercropping: Effects on soil nutrients, enzyme activities, and microbial community structure
Source: Front Plant Sci. 2023 Feb 20;14:1107690. doi: 10.3389/fpls.2023.1107690 (PMC9986260; doi:10.3389/fpls.2023.1107690)
Supplement: Supplementary file 1 [file DataSheet_1.docx]

Supplementary Figures


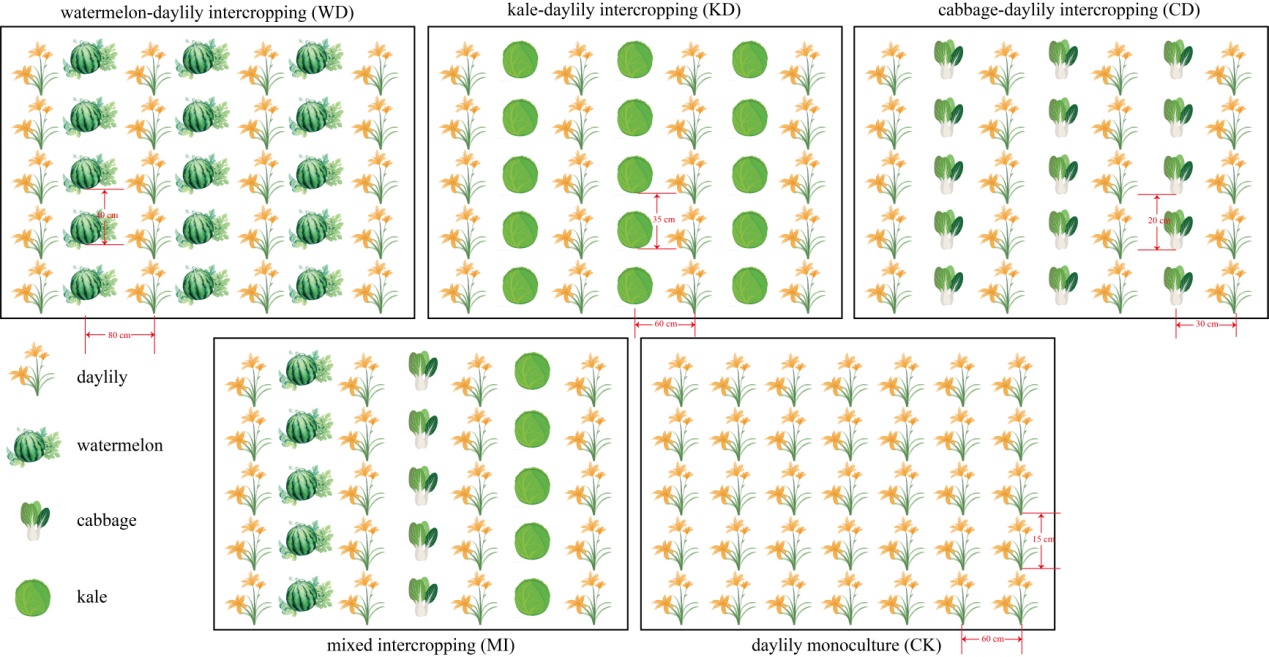


Figure S1. Planting framework of different intercropping systems. The spacing between watermelon plants was 40 cm, and the spacing between watermelon and daylily was 80 cm; the distance between cabbage plants was 20 cm, and the distance between cabbage and daylily was 30 cm; the distance between kale plants was 35 cm, and the distance between kale and daylily was 60 cm. In CK, the spacing between daylily plants was 15 cm, and the spacing between rows was 60 cm.


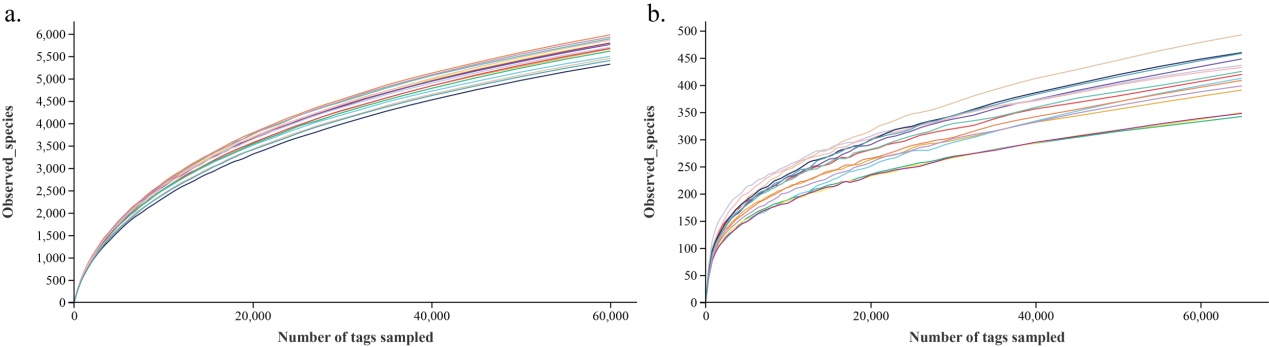


Figure S2. The rarefaction curve of the ITS2 region of fungi (a) and v3-v4 region of bacteria (b) in each sample under 97% similarity.
