# Supplementary material for: Daylily intercropping: Effects on soil nutrients, enzyme activities, and microbial community structure
Source: Front Plant Sci. 2023 Feb 20;14:1107690. doi: 10.3389/fpls.2023.1107690 (PMC9986260; doi:10.3389/fpls.2023.1107690)
Supplement: Supplementary file 2 [file Table_1.docx]

Supplementary tables

Table S1. ANOSIM and permutational MANOVA of different treatments effects on Soil microbial diversity

| Treatments | Bacteria | | | | Fungi | | | |
| --- | --- | --- | --- | --- | --- | --- | --- | --- |
|  | ANOSIM | | ADONIS | | ANOSIM | | ADONIS | |
|  | R | *P* | R^2^ | *P* | R | *P* | R^2^ | *P* |
| CD-vs-CK-vs-KD-vs-MI-vs-WD | 0.5674 | 0.001 | 0.5071 | 0.001 | 0.3778 | 0.006 | 0.4284 | 0.005 |

WD, watermelon-daylily intercropping; CD, cabbage-daylily intercropping; KD, kale-daylily intercropping; MI, row mixed intercropping, watermelon, cabbage, and kale were alternately intercropped with daylily; CK, monoculture daylily.

Table S2. Relative abundance of bacteria taxa from intercropping soils

| Level | Phylum | WD | CD | KD | MI | CK | *P*-value | Significant |
| --- | --- | --- | --- | --- | --- | --- | --- | --- |
| Phylum | *Proteobacteria* | 35.68±5.11a | 37.51±2.16a | 34.99±1.92a | 39.65±2.65a | 32.03±3.2a | 0.125 |  |
|  | *Gemmatimonadetes* | 16.33±2.69a | 17.28±1.02a | 16±0.79a | 15.6±0.95a | 17.28±1.53a | 0.605 |  |
|  | *Acidobacteria* | 10.06±1.02c | 12.01±1.84bc | 14.01±0.49ab | 13.57±1ab | 15.12±1.19a | 0.003 | ** |
|  | *Planctomycetes* | 8.39±3.97a | 9.69±1.49a | 11.53±1.49a | 7.02±0.63a | 12.17±1.83a | 0.07 |  |
|  | *Actinobacteria* | 6.91±1.72a | 6.19±0.37a | 6.63±0.63a | 6.38±0.34a | 7.76±0.66a | 0.306 |  |
|  | *Bacteroidetes* | 4.65±0.79ab | 4.26±0.44ab | 4.34±0.27ab | 5.85±1.1a | 3.56±0.28b | 0.02 | * |
|  | *Chloroflexi* | 3.44±0.53b | 4.07±0.18ab | 4.27±0.24a | 3.9±0.33ab | 4.41±0.04a | 0.026 | * |
|  | *Patescibacteria* | 1.74±0.46a | 2.11±0.69a | 2.41±0.09a | 2.36±0.46a | 1.56±0.25a | 0.147 |  |
|  | *Firmicutes* | 7.83±7.17a | 0.83±1.02a | 0.25±0.04a | 0.35±0.02a | 0.3±0.11a | 0.065 |  |
|  | *Rokubacteria* | 1.27±0.07ab | 1.34±0.24ab | 1.11±0.11b | 1.26±0.01ab | 1.56±0.18a | 0.044 | * |
| Genus | *Sphingomonas* | 2.82±0.44b | 3.83±0.39ab | 4.13±0.49a | 4.85±0.47a | 4.02±0.65ab | 0.008 | ** |
|  | *Lysobacter* | 3.01±0.97ab | 4.23±1.13a | 4.06±0.75a | 3.68±0.58a | 1.61±0.15b | 0.014 | * |
|  | *RB41* | 1.52±0.41c | 1.88±0.68bc | 2.93±0.26a | 2.71±0.32ab | 3.51±0.34a | 0.001 | ** |
|  | *Subgroup_10* | 1.17±0.1b | 1.42±0.11ab | 1.43±0.11ab | 1.57±0.19ab | 1.78±0.37a | 0.049 | * |
|  | *Lactobacillus* | 6.66±0.44a | ＜0.01b | ＜0.01b | ＜0.01b | ＜0.01b | 0 | ** |
|  | *MND1* | 1.22±0.56a | 1.2±0.15a | 1.33±0.12a | 1.25±0.12a | 1.67±0.14a | 0.289 |  |
|  | *SWB02* | 1.01±0.15a | 1.16±0.43a | 1.24±0.22a | 1.24±0.12a | 1.79±0.39a | 0.068 |  |
|  | *Dongia* | 0.78±0.19a | 0.88±0.35a | 0.81±0.14a | 0.84±0.12a | 1.18±0.18a | 0.222 |  |
|  | *SM1A02* | 0.76±0.33ab | 0.72±0.14ab | 0.78±0.04ab | 0.51±0.11b | 1.18±0.32a | 0.049 | * |
|  | *Arenimonas* | 0.56±0.16a | 0.66±0.43a | 0.72±0.22a | 0.86±0.18a | 0.49±0.07a | 0.444 |  |

Showen the top 10. Values are presented as mean ± standard error (n = 3). Different superscript letters indicate statistically significant differences (*P* < 0.05) by Tukey’s test between different treatments. *, indicates a significant difference at the P < 0.05 level; **, indicates a significant difference at the *P* < 0.01 level. WD, watermelon-daylily intercropping; CD, cabbage-daylily intercropping; KD, kale-daylily intercropping; MI, row mixed intercropping, watermelon, cabbage, and kale were alternately intercropped with daylily; CK, monoculture daylily.

Table S3. Relative abundance of fungi taxa from intercropping soils

| Level | Phylum | WD | CD | KD | MI | CK | *P*-value | Significant |
| --- | --- | --- | --- | --- | --- | --- | --- | --- |
| Phylum | *Ascomycota* | 86.26±4.19a | 87.87±6.15a | 88.35±5.52a | 87.9±5.05a | 95.09±2.55a | 0.271 |  |
|  | *Mortierellomycota* | 11±2.87a | 8.49±0.99ab | 5.44±2bc | 3.94±1.9bc | 1.54±0.54c | 0.001 | ** |
|  | *Basidiomycota* | 1.28±0.63c | 2.5±0.58c | 5.44±0.46b | 7.34±0.8a | 2.8±0.69c | 0 | ** |
|  | *Chytridiomycota* | 0.18±0.14a | 0.05±0.01a | 0.03±0.01a | 0.23±0.22a | 0.12±0.06a | 0.272 |  |
|  | *Mucoromycota* | 0.02±0.03b | 0.06±0.01a | ＜0.01b | 0.03±0.02ab | 0.01±0.02b | 0.021 | * |
|  | *Glomeromycota* | 0.01±0.01a | ＜0.01a | ＜0.01a | 0.08±0.07a | 0a | 0.069 |  |
|  | *Rozellomycota* | ＜0.01a | 0a | 0a | 0a | 0a | 0.452 |  |
|  | *Aphelidiomycota* | 0a | ＜0.01a | 0a | 0a | 0a | 0.076 |  |
| Genus | *Fusarium* | 7.68±0.51cd | 13.19±0.77b | 6.74±1.27d | 9.09±0.77c | 15.78±0.22a | 0 | ** |
|  | *Pseudaleuria* | 2.95±0.09c | 1.62±0.41d | 12.49±0.92a | 9.22±0.17b | 8.43±0.38b | 0 | ** |
|  | *Mortierella* | 11±1.7a | 8.49±0.98a | 5.44±2b | 3.94±0.19bc | 1.54±0.54c | 0 | ** |
|  | *Kotlabaea* | 5.77±0.98ab | 9.09±0.17a | 6.27±3.41ab | 4.91±0.35b | 1.1±0.22c | 0.002 | ** |
|  | *Schizothecium* | 5.44±1.12a | 5.72±0.39a | 6.84±1.06a | 5.24±0.95a | 2.16±0.45b | 0.001 | ** |
|  | *Coprinellus* | 0.35±0.38b | 0.09±0.02b | 4.49±0.52a | 5.51±1.03a | 0.1±0.13b | 0 | ** |
|  | *Peziza* | 0.11±0.18c | ＜0.01c | 0.02±0.03c | 0.93±0.19b | 8.32±0.48a | 0 | ** |
|  | *Madurella* | 3.79±0.09a | 0.1±0.02c | 0.28±0.16c | 0.29±0.05c | 2.8±0.45b | 0 | ** |
|  | *Acaulium* | 2.29±0.67a | 1.28±0.46ab | 0.38±0.06b | 0.63±0.27b | 1.01±0.68b | 0.006 | ** |
|  | *Microascus* | 0.97±0.13a | 1.04±0.38a | 1.01±0.16a | 0.65±0.05a | 0.87±0.14a | 0.208 |  |

Showen the top 10. Values are presented as mean ± standard error (n = 3). Different superscript letters indicate statistically significant differences (*P* < 0.05) by Tukey’s test between different treatments. *, indicates a significant difference at the *P* < 0.05 level; **, indicates a significant difference at the *P* < 0.01 level.

Table 4. Results of ANOVA like permutation test on the relationship between soil properties and microbial communities

| Variables | Bacteria | | Fungi | |
| --- | --- | --- | --- | --- |
|  | R^2^ | *P*-values | R^2^ | *P*-values |
| pH | 0.29 | 0.126 | 0.41 | 0.066 |
| OM | 0.85 | 0.001 | 0.17 | 0.339 |
| AN | 0.46 | 0.024 | 0.03 | 0.863 |
| AP | 0.44 | 0.035 | 0.41 | 0.025 |
| AK | 0.45 | 0.022 | 0.56 | 0.003 |
| UE | 0.78 | 0.001 | 0.06 | 0.715 |
| POD | 0.04 | 0.758 | 0.32 | 0.073 |
| SC | 0.71 | 0.001 | 0.64 | 0.001 |
| **Yield** | **0.44** | **0.027** | **0.06** | **0.691** |

AN, available nitrogen; AP, available phosphorus; AK, available potassium; OM, organic matter. UE, urease; POD, peroxidase; SC, sucrase.
